# Association between uric acid level and contrast-induced acute kidney injury in patients with type 2 diabetes mellitus after coronary angiography: a retrospective cohort study

**DOI:** 10.1186/s12882-022-03030-z

**Published:** 2022-12-12

**Authors:** Haixia Tang, Haoying Chen, Zuolin Li, Shengchun Xu, Gaoliang Yan, Chengchun Tang, Hong Liu

**Affiliations:** 1 Institute of Nephrology, Zhongda Hospital, Southeast University School of Medicine, Nanjing, Jiangsu China; 2 Department of Ultrasonography, Taizhou central hospital, Taizhou university hospital, Ningbo, China; 3 Department of Cardiology, Zhongda Hospital, Southeast University School of Medicine, Nanjing, Jiangsu China

**Keywords:** Uric acid, Contrast induced-acute kidney injury, Type 2 diabetes mellitus, Coronary angiology, Nomogram

## Abstract

**Background:**

This study assessed the predictive value of uric acid (UA) for contrast-induced acute kidney injury (CI-AKI) in patients with type 2 diabetes mellitus (T2DM) who underwent coronary angiography (CAG). A nomogram to aid in the prediction of CI-AKI was also developed and validated, and the construction of a prognostic nomogram combined with clinical features was attempted.

**Methods:**

This study retrospectively enrolled T2DM patients who underwent CAG between December 2019 and December 2020 at the Affiliated Zhongda Hospital of Southeast University. Multivariable logistic regression analysis was used for the analysis of clinical outcomes. Receiver operating characteristic (ROC) analyses were performed to determine the area under the ROC curve (AUC) and the cut-off points for continuous clinical data. The prediction accuracies of models for CI-AKI were estimated through Harrell’s concordance indices (C-index). Nomograms of the prognostic models were plotted for individualized evaluations of CI-AKI in T2DM patients after CAG.

**Results:**

A total of 542 patients with T2DM who underwent CAG were included in this study. We found that a high UA level (≥ 425.5 µmol/L; OR = 6.303), BUN level (≥ 5.98 mmol/L; OR = 3.633), Scr level (≥ 88.5 µmol/L; OR = 2.926) and HbA1C level (≥ 7.05%; OR = 5.509) were independent factors for CI-AKI in T2DM patients after CAG. The nomogram model based on UA, BUN, Scr and HbA1C levels presented outstanding performance for CI-AKI prediction (C-index: 0.878). Decision curve analysis (DCA) showed good clinical applicability in predicting the incidence of CI-AKI in T2DM patients who underwent CAG.

**Conclusion:**

High UA levels are associated with an increased incidence of CI-AKI in T2DM patients after CAG. The developed nomogram model has potential predictive value for CI-AKI and might serve as an economic and efficient prognostic tool in clinical practice.

## Introduction

Contrast-induced acute kidney injury (CI-AKI) is described as an acute decline in renal function following intravenous administration of contrast agents during angiography, such as enhanced computerized tomography (CT), enhanced magnetic resonance imaging (MRI), or vascular interventional treatment [1, 2]. The new diagnostic criterion proposed by the Kidney Disease: Improving Global Outcomes (KDIGO) guideline is an increased SCr level by ≥ 26.5 umol/l (0.3 mg/dl) within 48 h or by at least 50% compared to baseline values within one week after administration of the contrast agent [3] .Following the rapid development of interventional cardiology, CI-AKI has been a common complication of coronary angiography (CAG), which is associated with worse prognosis and is the third most common cause of hospital-acquired renal failure [4].

Type 2 diabetes mellitus (T2DM) has been proven to be a major risk factor for coronary heart disease [5]. Previous studies have shown that the T2DM population is at particularly high risk of CI-AKI [6, 7]. Despite improvements in management, CI-AKI remains associated with high morbidity and poor prognosis. There is currently no effective treatment for CI-AKI. Therefore, it is very important to identify patients at high risk and develop effective interventions.

Insulin resistance (IR) plays an important role in the pathophysiological process of T2DM [8]. Higher levels of serum insulin could promote renal reabsorption of uric acid (UA) and lead to UA injury [9]. Kuldeep Singh et al. reported that the incidence of hyperuricaemia in T2DM patients is 46% and that the prevalence is higher in women than in men [10]. Recently, the associations between UA and CI-AKI have received increasing attention. One study found that hyperuricaemia was significantly associated with the risk of CI-AKI in patients with relatively normal serum creatinine after percutaneous coronary interventions in China (OR = 5.83) [11]. Similar findings demonstrated that UA ≥ 8.0 mg/dL was associated with an increased risk of CI-AKI in patients receiving contrast-enhanced computerized tomography (CCT) [12]. In experimental models, hyperuricaemia was associated with an absence of intrarenal crystals, the manifestation of tubular injury, macrophage infiltration, and increased expression of inflammatory mediators [13].

Therefore, we aimed to investigate the predictive value of UA for the incidence of CI-AKI in patients with T2DM undergoing CAG and to build a predictive model of CI-AKI for clinical diagnosis and treatment.

## Methods

### Study population

We conducted a retrospective, observational, comparative cohort study at Affiliated Zhongda Hospital of Southeast University between December 2019 and December 2020. Study patients were older than 18 years with T2DM and suspected heart disease (with angina or chest pain and could not be diagnosed by electrocardiogram and echocardiography) who had undergone CAG. The criteria for excluding patients were as follows: (1) patients allergic to contrast; (2) Scr 442umol/L; (3) NYHA class IV patients who have unstable haemodynamics, and cannot lie for more than 24 h or use intra-aortic balloon counterpulsation; (4) patients who had undergone CT, MRI, or other contrast-assistant examinations within 14 days before participation in the study; (5) patients with blood pressure lower than 90/60mmHg or insufficient tissue perfusion; (6) patients with metabolic acidosis, severe infection or injuries, cancer, inflammatory diseases, or autoimmune diseases; (7) patients who had recently taken renal function-impairing medicines or suffered acute kidney injury. Patients with incomplete records of clinical data were also excluded.

This study was approved by the Clinical Research Ethics Committee of Affiliated Zhongda Hospital of Southeast University and informed consent was exempt duo to the retrospective nature of the study. (No: 2022ZDSYLL347-P01).

### Data collection and definitions

Data on demographic and clinical indicators were collected from electronic medical records of the Affiliated Zhongda Hospital of Southeast University. This information included sexy, age, systolic blood pressure (SBP) and diastolic blood pressure (DBP) at admission, previous medical and medicine history. All baseline laboratory tests were measured upon admission before the CAG or PCI. The postoperative Scr level was measured within 1 week after CAG or PCI and evaluated for determining the occurrence of CI-AKI. Hemoglobin (Hb) and platelet (PLT) were collected from routine blood tests. Total bilirubin (Tb), Blood Urea Nitrogen (BUN), Scr, uric acid (UA), triglyceride (TG), Total cholesterol (TC), high-density lipoprotein cholesterol (HDL-C), low-density lipoprotein cholesterol (LDL-C) and Hemoglobin A1C (HbA1C) were collected from blood biochemistry.

T2DM was defined as fasting blood-glucose (FBG) ≥ 7.0 mmol/L according to the American Diabetes Association’s standards of medical care [14] or self-reported history of T2DM or use of diabetes medications.

CI-AKI was diagnosed by the Kidney Disease: Improving Global Outcomes (KDIGO) guideline: An increase in SCr by ≥ 26.5 umol/l (0.3 mg/dl) within 48 h or to ≥ 1.5times baseline within one week after administration of the contrast agent [3].

The diagnosis of myocardial infarction (MI) was according to the Fourth Universal Definition of Myocardial Infarction [15].

The prophylactic hydration protocol used in our center was AMACING trial strategy [16].

### Statistical analysis

Statistical analysis was performed with SPSS 26.0 (IBM Corp., Armonk, NY, USA) and R version 4.0.2. Normally distributed data were expressed as the means ± standard deviation and were compared using independent sample t-tests. Data indicating poor normality were expressed as interquartile ranges, and rank-sum tests were used for the analysis. Continuous variables were analyzed by Student’s t-test or Mann-Whitney U-tests. Categorical variables were tested using Chi-square tests, or Fisher’s exact test when group numbers were small and the large number assumption for chi-square tests did not apply. Receiver operating characteristics (ROC) analyses were performed to determine the area under the ROC curve (AUC) and to determine the cutoff point of the continuous data. The AUCs were also provided with their sensitivity, specificity and 95% confidence intervals (CIs). A nomogram was created in the software package R using the nomogram function from the rms library. Validation of the nomogram included calibration and discrimination. Calibration was evaluated by calibration plots and Hosmer-Lemeshow tests to calculate the consistency between the observed and predicted probabilities. A Hosmer- Lemeshow P value > 0.05 indicated good consistency. The discrimination—namely, the predictive accuracy of a nomogram—was evaluated by the ROC curve. P values less than 0.05 were considered significant.

## Results

### Baseline characteristics of the CI-AKI and Non-CI-AKI groups

A total of 542 patients with T2DM who underwent CAG were included in this study. The incidence of CI-AKI was 9.4% (51/542) in this cohort. Table 1 shows the baseline characteristics of the CI-AKI and Non-CI-AKI groups. The results indicated that renal function factors, including BUN, Scr and UA were much higher in CI-AKI group than Non-CI-AKI group (*P* < 0.001, Table 1). In addition, patients with CI-AKI were older (*P* = 0.001) and more likely with hypertension (*P* = 0.005). The proportion of patients using β-blocker (*P* = 0.034) and digoxin (*P *< 0.001) differed between CI-AKI group and Non-CI-AKI group. Patients in CI-AKI group had lower level of Hb and higher level of HbA1C (*P *< 0.001). More importantly, patients in CI-AKI group were more likely to suffer from coronary heart disease which had more lesion vessels (*P* < 0.00).

**Table 1:** Baseline characteristics of the non-CI-AKI and CI-AKI groups

Variables	CI-AKI group *N* = 51	Non-CI-AKI group *N* = 491	*P* value
Male (n, %)	27 (52.9%)	280 (57.1%)	0.564
Age (years) P_50_ (P_25_–P_75_)	72 (62–76)	63 (56–71)	0.001
SBP (mmHg) P_50_ (P_25_–P_75_)	136 (122–155)	134 (122–146)	0.222
DBP (mmHg) P_50_ (P_25_–P_75_)	78 (69–84)	76 (69–84)	0.799
BMI (kg/m^2^) P_50_ (P_25_–P_75_)	25 (23–27)	25 (23–27)	0.545
Hypertension (n, %)	47 (92.2%)	348 (71.0%)	0.005
Atrial fibrillation (n, %)	6 (11.8%)	33 (6.7%)	0.186
Acute myocardial infarction (n, %)	7 (13.7%)	38 (7.8%)	0.142
Coronary angiology			< 0.001
Lesion vessels (N ≤ 2)	26 (51.0%)	363 (73.9%)	
Lesion vessels (N ≥ 3)	25 (49.0%)	128 (26.1%)	
Dose of contrast agent P_50_ (P_25_–P_75_) (ml)	35 (25–100)	30 (20–60)	0.069
Hydration (n, %)	51 (100%)	489 (99.8%)	0.747
In-hospital medication			
Aspirin (n, %)	45 (85.2%)	419 (85.5%)	0.596
ACEI/ARB (n, %)	30 (58.8%)	232 (47.4%)	0.119
β-blocker (n, %)	39 (76.5%)	301 (61.4%)	0.034
Statin (n, %)	48 (94.1%)	456 (93.1%)	0.776
Digoxin (n, %)	7 (13.7%)	14 (2.9%)	0.000
Laboratory test			
Hb P_50_ (P_25_–P_75_)	125 (112–137)	138 (128–150)	0.000
PLT P_50_ (P_25_–P_75_)	188 (160–244)	201 (166–236)	0.613
TB P_50_ (P_25_–P_75_)	10.5 (7.6–15.5)	11.2 (9.0–14.1)	0.613
BUN P_50_ (P_25_–P_75_)	7.5 (6.0–9.4)	5.4 (4.6–6.5)	0.000
Scr P_50_ (P_25_–P_75_)	103 (67–153)	71 (61–82)	0.000
UA P_50_ (P_25_–P_75_)	464 (321–599)	325 (280–383)	0.000
TG P_50_ (P_25_–P_75_)	1.42 (1.04–2.43)	1.34 (0.97–1.96)	0.225
TC P_50_ (P_25_–P_75_)	4.20 (3.35–5.11)	4.21 (3.43–4.97)	0.616
HDL-C P_50_ (P_25_–P_75_)	1.11 (0.96–1.39)	1.12 (0.95–1.31)	0.137
LDL-C P_50_ (P_25_–P_75_)	2.75 (2.33–3.22)	2.47 (1.91–3.13)	0.315
HbA1C P_50_ (P_25_–P_75_)	7.6 (6.5–8.7)	6.3 (5.7–7.5)	0.000

*SBP* systolic blood pressure, *DBP* diastolic blood pressure, *BMI*, body mass index, *ACEI/ARB* angiotensin-converting enzyme inhibitors/ angiotensin receptor blockers, *Hb* hemoglobin, *PLT* platelets, *TB* total bilirubin, *BUN* blood urea nitrogen, *Scr*, serum creatinine, *UA* uric acid, *TG* triglyceride, *TC*, total cholesterol, *HDL-C* high-density lipoproteincholesterol, *LDL-C* low-density lipoprotein cholesterol, *HbA1C* glycosylated hemoglobin

### Determining the cutoff values

**Fig. 1:**
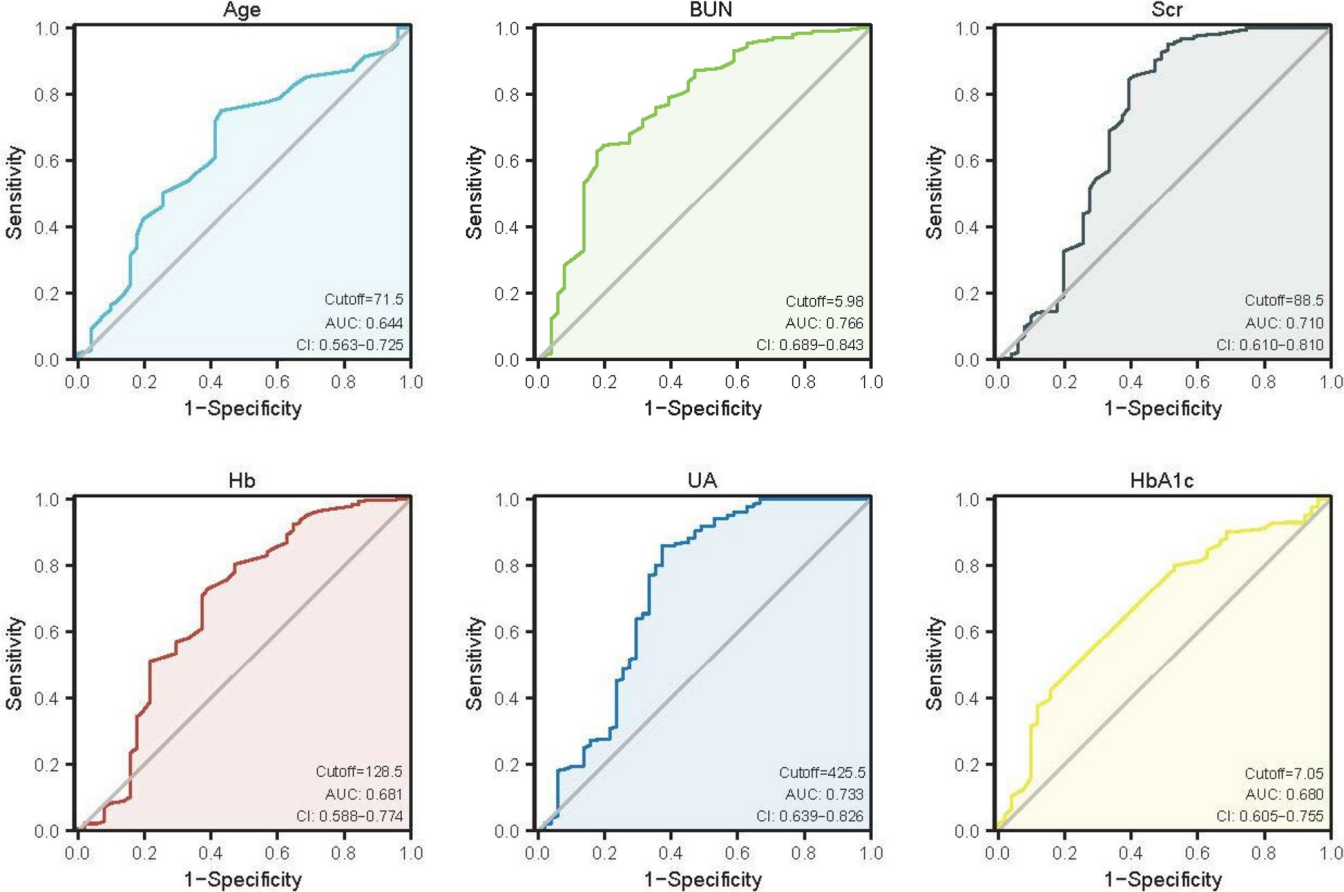
The ROC curve. The ROC curve for Age, BUN, Scr, Hb, UA and HbA1c. The cutoff values for these indicators were identified. *P* < 0.05 indicates significant differences

We then used the ROC curves (Fig. 1) to calculate optimal cutoff values of continuous data based on Youden’s index. The cutoff values for statistically significant CI-AKI related indictors, including age, BUN, Scr, Hb, UA and HbA1C were identified.

### Independent risk factors for CI-AKI in T2DM patients

**Fig. 2:**
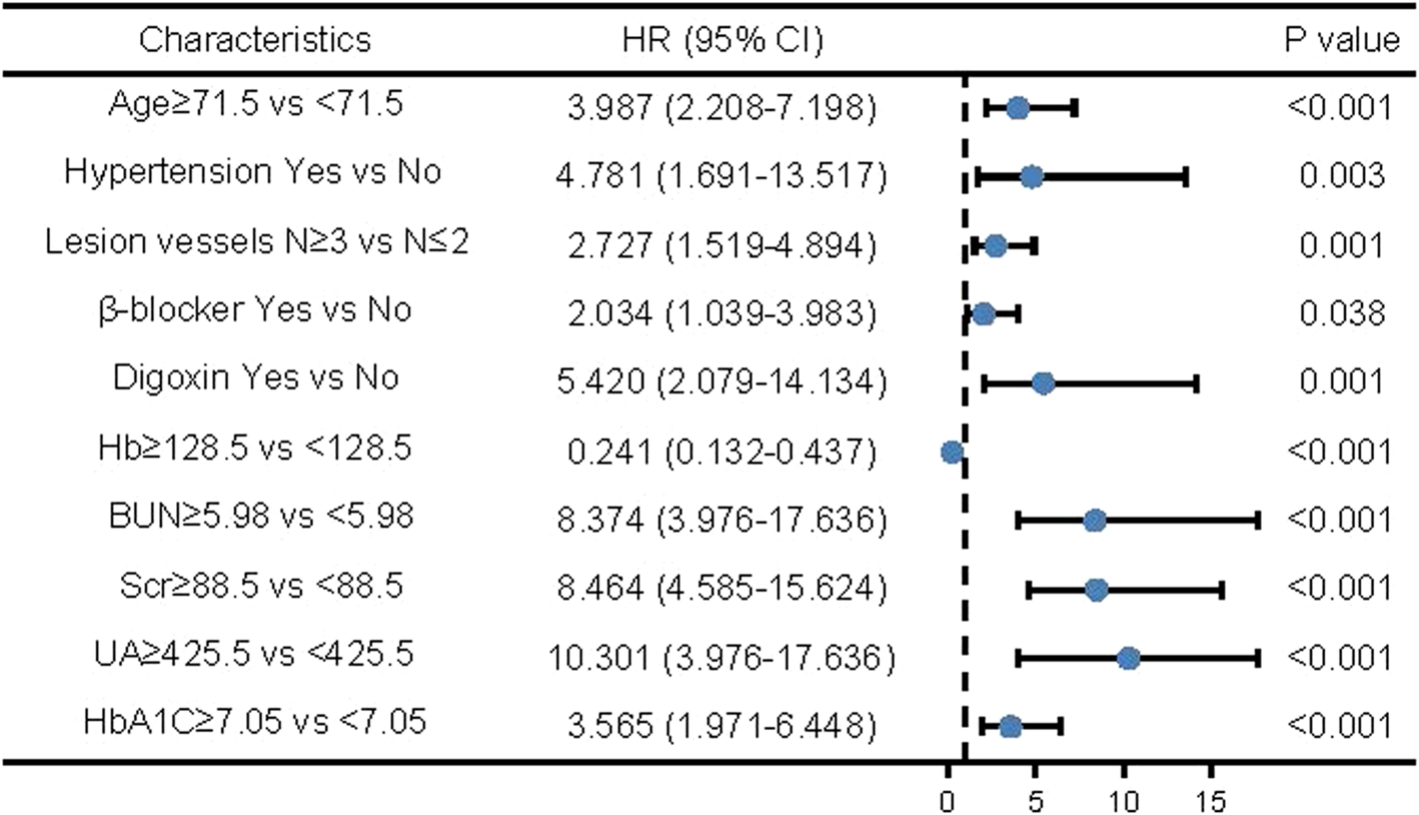
Univariate logistic regression analyses for CI-AKI. *P* < 0.05 indicates significant differences

**Fig. 3:**
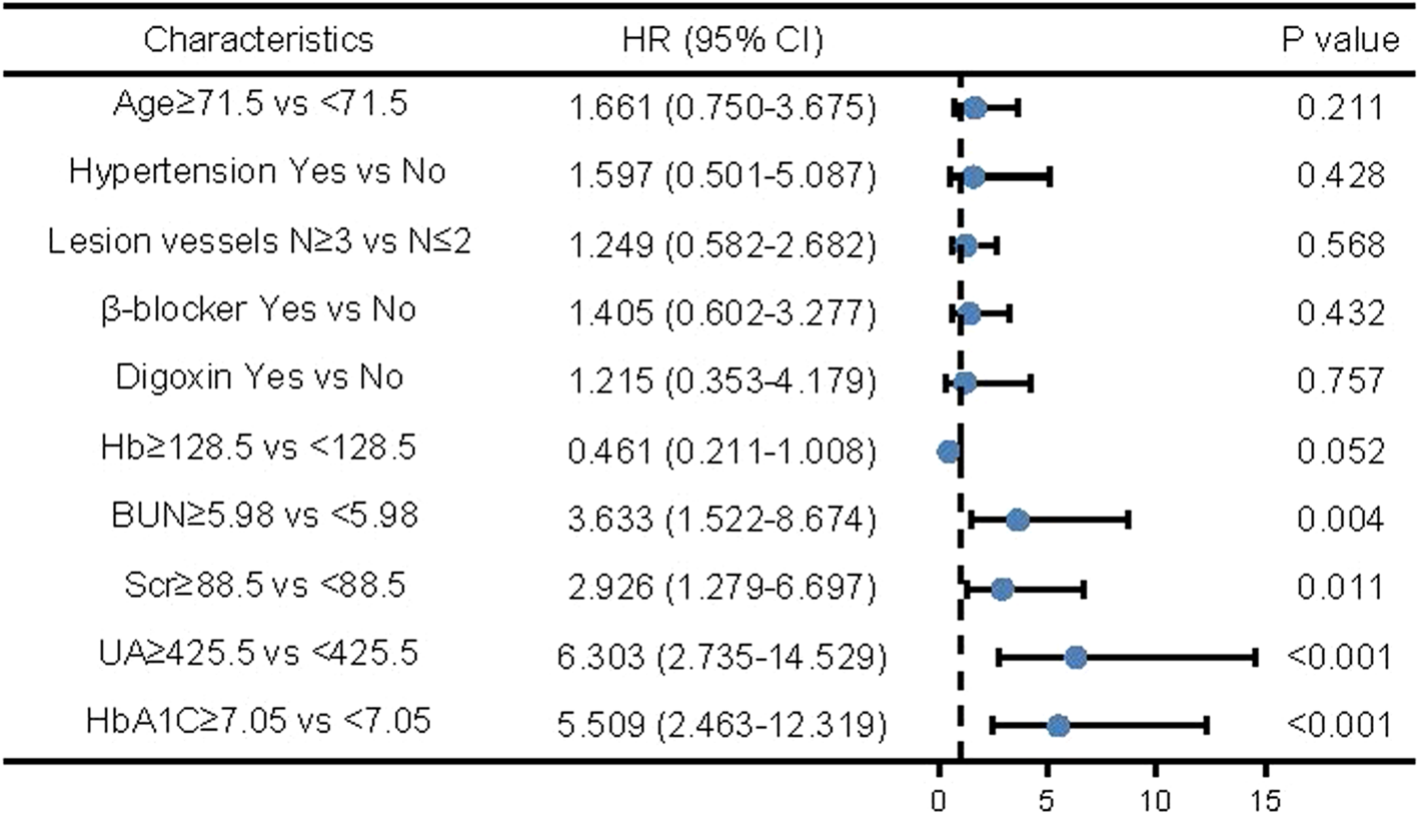
Multivariate logistic regression analyses for LNM. *P* < 0.05 indicates significant differences

All baseline characteristics and laboratory tests were analyzed by univariate and multivariate logistic regression analyses in T2DM patients. The results of univariate logistic regression analysis showed that age, hypertension, lesion vessels, β-blocker, digoxin, Hb, BUN, Scr, UA and HbA1C were associated with CI-AKI (all *P* < 0.05, Fig. 2). Then, significant factors (*P* < 0.1) from the univariate analysis were included in the multivariate analysis. The results of multivariate analysis showed that BUN, Scr, UA and HbA1C were independent factors for CI-AKI (all *P* < 0.05, Fig. 3).

### Building and validating the nomogram

**Fig. 4:**
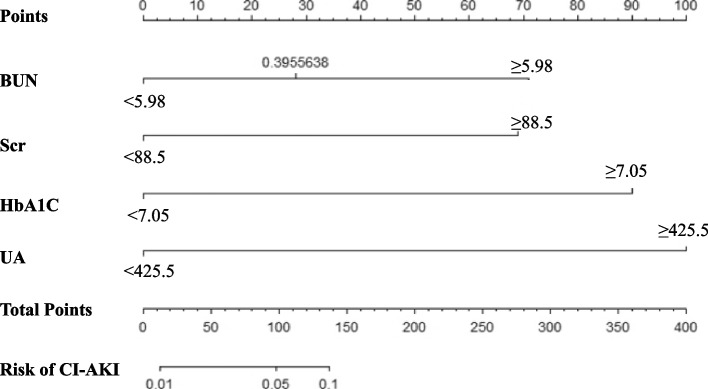
The nomogram for predicting CI-AKI with BUN, Scr, HbA1C and UA

**Fig. 5:**
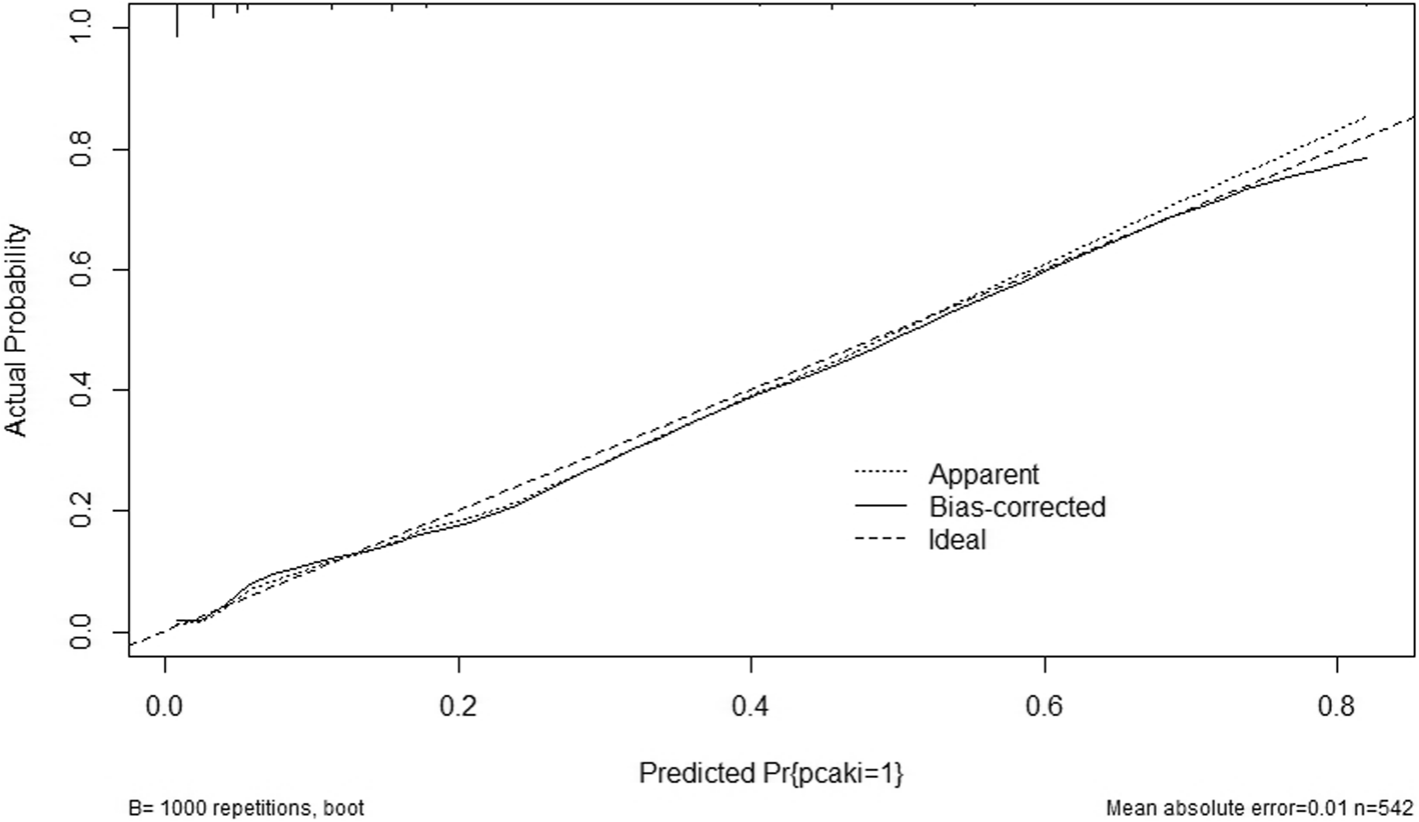
The calibration plots for the nomogram

**Fig. 6:**
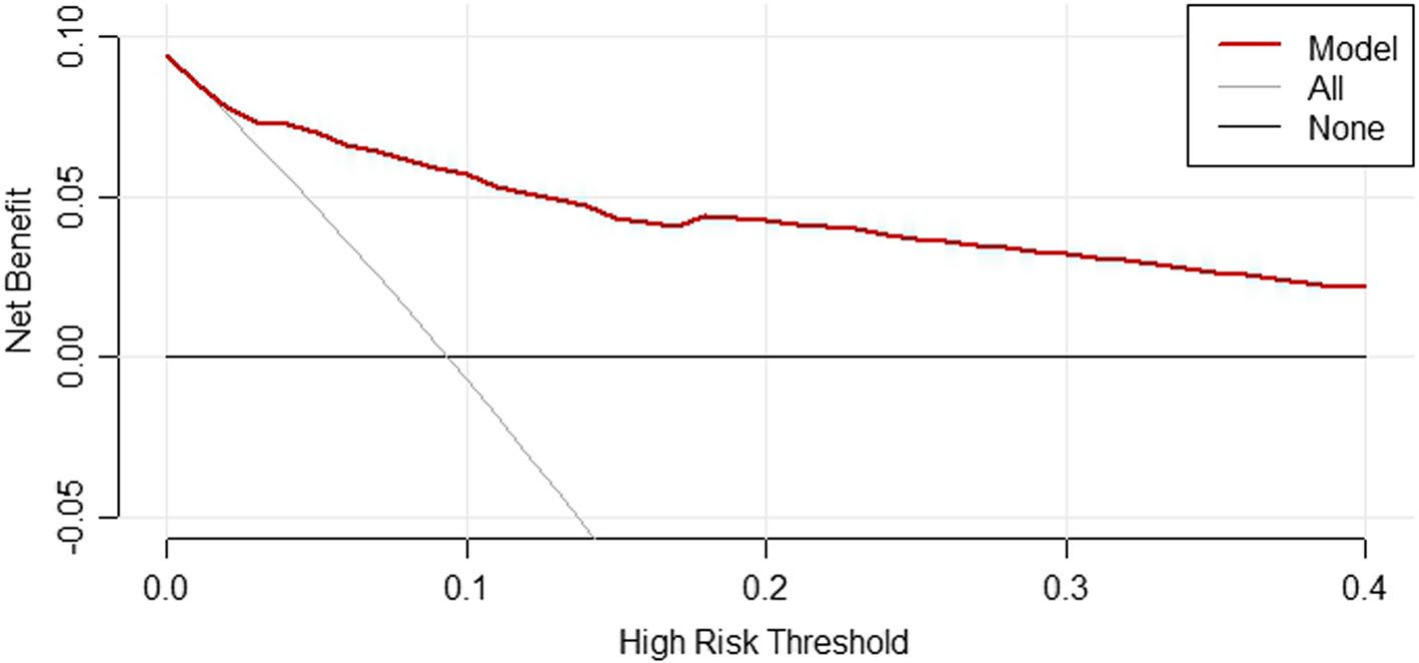
Decision curve analysis of the nomogram

Finally, four statistically significant prognostic factors (BUN, Scr, UA and HbA1C) from multivariate logistic regression analyses model were integrated to nomogram (Fig. 4). For each patient, four lines were drawn upward to determine the points received from the four predictors in the nomogram. The sum of these points was located on the Total Points axis which could be used to determine the possibility of CI-AKI in T2DM patients who underwent coronary angiology (Fig. 4). The C-index of this model was 0.878, which showed the good discrimination ability of the model. The calibration curve of the incidence of CI-AKI in T2DM patients was close to the ideal diagonal line, indicating good consistency between the predicted value and the actual observed value (Fig. 5). In addition, decision curve analysis (DCA) was used to prove the clinical usefulness for our nomogram model. Results showed good clinical applicability of our model in predicting the incidence of CI-AKI in T2DM patients who underwent coronary angiology (Fig. 6).

## Discussion

CI-AKI is a common iatrogenic complication associated with increased health resources and utilization and adverse outcomes, including short- and long-term mortality and accelerated progression of preexisting renal insufficiency [17]. Previous studies have confirmed that CI-AKI is independently associated with adverse events, including death, MI, and bleeding, the rates of which dramatically increase if RRT is needed, whether temporary or permanent [18]. For these reasons, considerable efforts should be devoted to identifying groups at high risk of CI-AKI and preventing that type of injury in high-risk groups. T2DM is a clear independent risk factor for CI-AKI. T2DM primarily occurs because of defects in insulin secretion and insulin resistance [19]. Our previous work proved that high insulin resistance (measured by the triglyceride-glucose index) was associated with an increased incidence of CI-AKI [20]. This study also showed that T2DM patients with high UA levels (≥ 425.5 µmol/L) had a significantly increased incidence of CI-AKI.

Early studies proved that preexisting renal disease, elderly people, DM, congestive heart failure, hypovolemic status, administration of nephrotoxic agents, and a large amount of contrast medium were all associated with CI-AKI [21, 22]. Among them, preexisting chronic kidney disease is the strongest patient-related risk factor, with lower levels of kidney function associated with higher degrees of risk [23]. The levels of Scr and BUN can indicate kidney function. In our study, we found that high Scr levels (≥ 88.5 µmol/L) and high BUN levels (≥ 5.98 mmol/L) were both independent risk factors for the incidence of CI-AKI in T2DM patients after CAG. However, Scr concentration has a number of limitations as a biomarker of AKI, not least that it is affected by a number of factors other than renal function, there is a delay before it rises after renal injury, and as a functional marker, it does not provide information about the nature or aetiology of renal damage [24]. For these reasons, we thought that individual Scr or BUN could not reflect the true risk of CI-AKI in T2DM patients after CAG and tried to build a nomogram model to predict the incidence of CI-AKI in T2DM patients.

UA is a major antioxidant in the body [13]. Serum UA, that is, in concentrations that do not cause crystal precipitations, has been associated with hypertension, chronic kidney disease, cardiovascular diseases, stroke, diabetic nephropathy, metabolic syndrome and acute kidney injury. Previous studies have proven that uric acid level was independently associated with an increased risk of contrast-induced nephropathy after CAG [25, 26]. We found that a high UA level (≥ 425.5 µmol/L) was an independent risk factor for CI-AKI in T2DM patients who underwent CAG (HR = 6.303, 95% CI = 2.735–14.529, *P* < 0.001). Previous reports obtained similar findings that elevated serum UA was associated with CA-AKI after reperfusion in patients with ST elevation myocardial infarction (STEMI) treated with PCI (CA-AKI 25%: odds ratio 1.32, 95% CI 1.03–1.69, *p* = 0.027; CA-AKI 0.5: odds ratio 1.76, 95% CI 1.11–2.79, *p* = 0.016) [27]. Hyperuricaemia was significantly associated with the risk of CI-AKI in patients with relatively normal serum creatinine after percutaneous coronary interventions (OR = 5.38, 95% CI = 1.99–14.58, *P* = 0.001) [11]. The possible relationship between UA and CI-AKI may be associated with an increase in vasoconstrictive and oxidative agent synthesis and with a greater inflammatory response after CAG [28–30]. UA has been proven to inactivate and inhibit the release of nitric oxide from endothelial cells and to simultaneously increase endothelin 1 (ET-1), leading to vasoconstriction [31, 32]. Moreover, in the setting of STEMI, in which transitory cardiac output reduction can cause renal hypoperfusion and ischaemia, UA may potentially contribute to ischaemia‒reperfusion injury and thus to the development of CA-AKI by enhancing oxidative stress, inflammation and endothelial dysfunction [27]. In this study, we also found that a high HbA1c level (≥ 7.05%) was associated with an increased incidence of CI-AKI in T2DM patients after CAG. The HbA1c level reflects the average glucose over the preceding eight to twelve weeks of glycaemic control and is viewed as a more accurate and stable measure than fasting blood glucose level [33]. High HbA1c was associated with poor glycaemic control and with a high risk for kidney disease progression in some previous studies [34].

Finally, we constructed a prognostic model for CI-AKI in T2DM patients who underwent CAG based on renal function (UA, BUN and Scr) and blood glucose level (HbA1C). The C-index of this model was 0.878, which showed the good discrimination ability of the model. The calibration curve of the incidence of CI-AKI in T2DM patients was close to the ideal diagonal line, indicating good consistency between the predicted value and the actual observed value. We believe that this model has good clinical applicability for risk stratification in the contemporary real-world management of CI-AKI.

### Study limitations

Our study has the following limitations. First, it was a retrospective study with small total number of cases in single center. Second, the main drawback was the lack of internal or external validation. Therefore, multicenter studies with larger sample sizes are needed for external validation of our nomogram model.

## Conclusion

In conclusion, the results of our study confirm that an elevated UA level is an independent risk factor for CI-AKI in T2DM patients after CAG. In addition, a nomogram model (UA, Scr, BUN and HbA1c levels) is helpful for early prediction and may aid in the prevention of CI-AKI in T2DM patients after CAG.

## Data Availability

The datasets analysed during this study are obtainable from the corresponding author upon reasonable request.

## Abbreviations

UA: Uric acid
CI-AKI: Contrast-induced acute kidney injury
T2DM: Type 2 diabetes mellitus
CAG: Coronary angiography
ROC: Receiver operating characteristic
AUC: Area under the ROC curve
C-index: Concordance indices
CT: Computerized tomography
MRI: Enhanced magnetic resonance imaging
IR: Insulin resistance
SBP: Systolic blood pressure
DBP: Diastolic blood pressure
BMI: Body mass index
ACEI/ARB: Angiotensin-converting enzyme inhibitors/ angiotensin receptor blockers
Hb: Hemoglobin
PLT: Platelets
TB: Total bilirubin
BUN: Blood urea nitrogen
Scr: Serum creatinine
TG: Triglyceride
TC: Total cholesterol
HDL-C: High-density lipoproteincholesterol
LDL-C: Low-density lipoprotein cholesterol
HbA1C: Glycosylated hemoglobin

## References

[1] Azzalini L, Candilio L, McCullough P, Colombo A (2017). Current risk of Contrast-Induced Acute kidney Injury after coronary angiography and intervention: a reappraisal of the literature. Can J Cardiol.

[2] Kooiman J, van de Peppel W, Sijpkens Y, Brulez H, de Vries P, Nicolaie M, Putter H, Huisman M, van der Kooij W, van Kooten C (2015). No increase in kidney Injury Molecule-1 and Neutrophil Gelatinase-Associated Lipocalin excretion following intravenous contrast enhanced-CT. Eur Radiol.

[3] Khwaja A (2012). KDIGO clinical practice guidelines for acute kidney injury. Nephron Clin Pract.

[4] Liu J, Sun G, He Y, Song F, Chen S, Guo Z, Liu B, Lei L, He L, Chen J (2019). Early β-blockers administration might be associated with a reduced risk of contrast-induced acute kidney injury in patients with acute myocardial infarction. J Thorac disease.

[5] Song L, Zhang D, Guo C, Gu Z, Wang L, Yao Y, Wang H, Zeng Z, Wang W, Yang Y (2021). The traditional chinese medicine formula Fufang-Zhenzhu-Tiaozhi protects myocardia from injury in diabetic minipigs with coronary heart disease. Biomed Pharmacother.

[6] Gami A, Garovic V (2004). Contrast nephropathy after coronary angiography. Mayo Clin Proceedings.

[7] Toprak O (2007). Risk markers for contrast-induced nephropathy. Am J Med Sci.

[8] Li Y, Liu Y, Shi D, Yang L, Liang J, Zhou Y (2016). Insulin resistance increases the risk of Contrast-Induced Nephropathy in Patients undergoing elective coronary intervention. Angiol.

[9] Gaubert M, Bardin T, Cohen-Solal A, Diévart F, Fauvel  J, Guieu R, Sadrin S, Maixent J, Galinier M, Paganelli F (2020). Hyperuricemia and hypertension, coronary artery disease, kidney disease: from Concept to Practice. Int J Mol Sci.

[10] Singh K, Kumar P, Joshi A, Shivhare D, Mahto S, Singh A, Aneja A, Lamba B (2019). Study of association of serum uric acid with albuminuria and carotid atherosclerosis in type 2 diabetes mellitus patients. J family Med Prim care.

[11] Liu Y, Tan N, Chen J, Zhou Y, Chen L, Chen S, Chen Z, Li L (2013). The relationship between hyperuricemia and the risk of contrast-induced acute kidney injury after percutaneous coronary intervention in patients with relatively normal serum creatinine. Clin (Sao Paulo Brazil).

[12] Wu M, Tsai S, Lee C, Wu C (2019). The predictive value of Hyperuricemia on Renal Outcome after contrast-enhanced computerized tomography. J Clin Med.

[13] Ejaz A, Johnson R, Shimada M, Mohandas R, Alquadan K, Beaver T, Lapsia V, Dass B (2019). The role of Uric Acid in Acute kidney Injury. Nephron.

[14] American Diabetes Association. 15. Diabetes Care in the Hospital: Standards of Medical Care in Diabetes-2021. Diabetes Care. 2021;44(Suppl 1):S211-20. 10.2337/dc21-S015 33298426

[15] Thygesen K, Alpert J, Jaffe A, Chaitman B, Bax J, Morrow D, White H (2018). Fourth Universal Definition of myocardial infarction (2018). Circulation.

[16] Nijssen E, Rennenberg R, Nelemans P, Essers B, Janssen M, Vermeeren M, Ommen V, Wildberger J (2017). Prophylactic hydration to protect renal function from intravascular iodinated contrast material in patients at high risk of contrast-induced nephropathy (AMACING): a prospective, randomised, phase 3, controlled, open-label, non-inferiority trial. Lancet (London, England).

[17] Hossain M, Costanzo E, Cosentino J, Patel C, Qaisar H, Singh V, Khan T, Cheng J, Asif A, Vachharajani T (2018). Contrast-induced nephropathy: pathophysiology, risk factors, and prevention. Saudi J kidney Dis Transpl.

[18] Ali Z, Karimi Galougahi K, Nazif T, Maehara A, Hardy M, Cohen D, Ratner L, Collins M, Moses J, Kirtane A (2016). Imaging- and physiology-guided percutaneous coronary intervention without contrast administration in advanced renal failure: a feasibility, safety, and outcome study. Eur Heart J.

[19] Karalliedde J, Gnudi L (2016). Diabetes mellitus, a complex and heterogeneous disease, and the role of insulin resistance as a determinant of diabetic kidney disease. Nephrol Dial Transplant.

[20] Qin Y, Tang H, Yan G, Wang D, Qiao Y, Luo E, Hou J, Tang C (2020). A high triglyceride-glucose index is associated with contrast-induced acute kidney injury in Chinese patients with type 2 diabetes mellitus. Front Endocrinol.

[21] Mehran R, Dangas G, Weisbord S (2019). Contrast-Associated Acute kidney Injury. N Engl J Med.

[22] Silver S, Shah P, Chertow G, Harel S, Wald R, Harel Z (2015). Risk prediction models for contrast induced nephropathy: systematic review. BMJ (Clinical research ed).

[23] McCullough P, Adam A, Becker C, Davidson C, Lameire N, Stacul F, Tumlin J (2006). Risk prediction of contrast-induced nephropathy. Am J Cardiol.

[24] Selby N, Fluck R, Kolhe N, Taal M (2016). International Criteria for Acute kidney Injury: advantages and remaining Challenges. PLoS Med.

[25] Barbieri L, Verdoia M, Schaffer A, Cassetti E, Marino P, Suryapranata H, De Luca G (2015). Uric acid levels and the risk of contrast Induced Nephropathy in patients undergoing coronary angiography or PCI. Nutr Metab Cardiovas Dis: NMCD.

[26] Saritemur M, Turkeli M, Kalkan K, Tanboga İH, Aksakal E (2014). Relation of uric acid and contrast-induced nephropathy in patients undergoing primary percutaneous coronary intervention in the ED. Am J Emerg Med.

[27] Mandurino-Mirizzi A, Kajana V, Cornara S, Somaschini A, Demarchi A, Galazzi M, Crimi G, Ferlini M, Camporotondo R, Gnecchi M (2021). Elevated serum uric acid is a predictor of contrast associated acute kidney injury in patient with ST-segment elevation myocardial infarction undergoing primary percutaneous coronary intervention. Nutr Metab Cardiovasc Dis: NMCD.

[28] Ho W, Tsai W, Yu K, Tsay P, Wang C, Hsu T, Kuo C (2010). Association between endothelial dysfunction and hyperuricaemia. Rheumatol (Oxford).

[29] Mandurino-Mirizzi A, Demarchi A, Ruffinazzi M, Cornara S, Somaschini A, Crimi G, Ferlini M, Camporotondo R, Gnecchi M, Ferrario M (2020). Serum uric acid may modulate the inflammatory response after primary percutaneous coronary intervention in patients with ST-elevation myocardial infarction. J Cardiovasc Med (Hagerstown Md).

[30] Tanık V, Çınar T, Velibey Y, Öz A, Kalenderoğlu K, Gümüşdağ A, Aruğaslan E, Keskin M, Eren M (2019). Neutrophil-to-lymphocyte ratio predicts Contrast-Induced Acute kidney Injury in Patients with ST-Elevation myocardial infarction treated with primary percutaneous coronary intervention. J Tehran Heart Cent.

[31] McCullough P, Choi J, Feghali G, Schussler J, Stoler R, Vallabahn R, Mehta A (2016). Contrast-induced acute kidney injury. J Am Coll Cardiol.

[32] Pistolesi V, Regolisti G, Morabito S, Gandolfini I, Corrado S, Piotti G, Fiaccadori E (2018). Contrast medium induced acute kidney injury: a narrative review. J Nephrol.

[33] Lin C, Yang C, Li C, Liu C, Chen C, Lin W, Hwang K, Yang S, Li T (2014). Visit-to-visit variability of fasting plasma glucose as predictor of ischemic stroke: competing risk analysis in a national cohort of Taiwan Diabetes Study. BMC Med.

[34] Navaneethan S, Schold J, Jolly S, Arrigain S, Winkelmayer W, Nally J (2017). Diabetes control and the risks of ESRD and mortality in patients with CKD. Am J kidney Dis.

